# Positional Accuracy of Dental Implants Placed by Means of Fully Guided Technique in Partially Edentulous Patients: A Retrospective Study

**DOI:** 10.1002/cre2.70144

**Published:** 2025-05-19

**Authors:** Mariano Tia, Alessia Teresa Guerriero, Antonio Carnevale, Ilaria Fioretti, Gianrico Spagnuolo, Gilberto Sammartino, Roberta Gasparro

**Affiliations:** ^1^ Department of Neuroscience, Reproductive Science and Dentistry University of Naples Federico II Naples Italy

**Keywords:** dental implant, digital implant, guided surgery, implant surgery, retrospective study

## Abstract

**Objectives:**

Computer‐aided implant surgery (CAIS) is a fully digital approach that guides the biological and prosthetic ideal implant position. The aim of this retrospective clinical study was to assess the accuracy of implant position using CAIS and clinical outcomes, in partially edentulous patients.

**Material and Methods:**

This study was designed as retrospective study. Twenty‐one patients requiring a maximum of two implants were recruited from 2023 to 2024 at the University of Naples Federico II. For all patients, 3D cone‐beam computed tomography (CBCT) and intraoral scans were obtained and superimposed by matching the resulting DICOM and STL data files in a software to create the tooth‐supported surgical guide. All implants were placed using a fully guided implant surgery protocol. The accuracy of the technique was measured by the deviation between the actual implant position (mesio‐distal deviation, depth error, and axis deviation) obtained from the postoperative CBCT and the preoperative planned implant position. Clinical outcomes assessed included biological complications, implant and prosthetic failures, esthetic outcomes, and patient satisfaction. Descriptive analysis was performed using mean and standard deviation.

**Results:**

A total of 37 implants were analyzed. The mean results were as follows: 0.43 ± 0.20 mm of mesio‐distal linear deviation at the implant shoulder, 0.24 ± 0.07 mm of depth error, and 1.46° ± 0.31° of axis deviation. At 6 months, healing was uneventful for all patients, with no complications or implant or prosthetic failures reported. Patients with implant‐supported restorations expressed high levels of functional and esthetic satisfaction.

**Conclusions:**

The fully guided technique achieved clinically acceptable accuracy positioning of dental implants in partially edentulous patients.

## 1 Introduction

The increase in life expectancy among the global population, combined with technological advances in modern dentistry, has led to a growing demand for implant‐prosthetic rehabilitations as the preferred treatment for partial or total edentulism (Ducommun et al. 2019). Moreover, properly positioned and osseointegrated implants ensure the long‐term success of implant‐prosthetic rehabilitations from both functional and esthetic perspectives (Brånemark et al. 1977). The success of these rehabilitations can be compromised by early or late complications, such as infections, lack of primary stability, and damage to vital anatomical structures (e.g., the mandibular neurovascular bundle, maxillary sinus, and nasal cavity), which may result in paresthesia or bleeding, as well as bone and soft tissue dehiscences that can expose implant components (Misch et al. 2008; Widmann and Bale 2006). Additional complications, primarily of biomechanical origin, relate to the alignment of the implant with the prosthetic device (e.g., fractures of the implant, screw connections, or abutments) (Sailer et al. 2022). Thus, the key to a successful implant‐prosthetic restoration lies in the precise placement of the implant, considering not only the anatomy of the surrounding soft and hard tissues but also the design of the subsequent dental prosthesis (Evans and Chen 2008; Brief et al. 2005). Achieving this requires a thorough clinical analysis of the patient's individual anatomy and meticulous preoperative planning of the implant position, guided by prosthetic considerations (Katafuchi et al. 2018). The precision of implant planning is enhanced when assisted by computer technology. Digital tools can be employed to program the insertion of intraosseous implants, ensuring that the implant‐prosthetic rehabilitation achieves optimal esthetic and functional outcomes. This process, known as computer‐aided implant surgery (CAIS), can be executed using either a fully digital or partially digital workflow (Tahmaseb et al. 2018; Hämmerle et al. 2009). Numerous studies have shown that guided implantology provides greater accuracy in implant placement compared to free‐hand techniques, with fully guided approaches proving to be more accurate than partially guided ones (Guentsch et al. 2021; Tattan et al. 2020; Raico Gallardo et al. 2017; Zhou et al. 2018); on the other hand, it should be noted that the guided technique has some drawbacks, such as higher costs compared to traditional implant methods and a learning curve for the surgeon (Pozzi et al. 2014a). Additionally, the use of a surgical template can limit the flow of cooling fluids to the drill, which is in direct contact with the bone, potentially increasing bone temperature. This rise in temperature could negatively affect the process of osseointegration (Li et al. 2019a).

The fully digital guided approach begins with the acquisition of a cone beam computed tomography (CBCT) scan to generate high‐resolution three‐dimensional reconstructions of the maxillary bone structures, which are stored as DICOM files (Digital Imaging and Communications in Medicine) (Jung et al. 2009; Bornstein et al. 2014). Subsequently, intraoral scans are used to create STL (Standard Tessellation Language) files, which provide information about the surface of the edentulous area (Lee et al. 2015). This information is transferred to virtual imaging software, where the files are matched to create a 3D model of the patient's soft and hard tissues, facilitating the planning of individualized implant treatment. The design of the implant‐prosthetic restoration is then carried out on this virtual model using Computer‐Aided Design (CAD) software, allowing for the virtual simulation of the three‐dimensional implant insertion and the identification of both the spatial orientation and ideal size of the implants based on the chosen prosthetic treatment (D'haese et al. 2017). The spatial coordinates for implant positioning relative to the surgical site are used to produce a surgical template via Computer‐Aided Manufacturing (CAM) with 3D printers. During the intraoperative phase, the surgical template is stabilized on the jaw (depending on the type of support: bone, dental, mucosal, or mixed) to guide the surgeon in the correct preparation of the implant site using a dedicated drill kit and in the precise positioning of the implants in the edentulous sites (Pettersson et al. 2014a; Tahmaseb et al. 2014). Each step in the CAIS process carries the potential for errors that could compromise proper implant placement. Factors related to the patient (e.g., limited mouth opening, presence, or absence of dental support for the surgical template, sudden movements during the procedure), the type of surgical template support, the choice of an open‐flap or flapless approach, and the experience of the operator can all affect the accuracy of implant placement (Tatakis et al. 2019; Gargallo‐Albiol et al. 2022). Anyway, research indicates that implants placed using flapless computer‐guided techniques have an average survival rate of 97% over a follow‐up period of approximately 22.6 months (Moraschini et al. 2015), and studies comparing flapless implant placement with traditional computer‐guided techniques have found no significant differences in implant survival rates between the two methods (Yogui et al. 2021). The guided technique has some drawbacks, such as higher costs compared to traditional implant methods and a learning curve for the surgeon (Pozzi et al. 2014); additionally, the use of a surgical template can limit the flow of cooling fluids to the drill, which is in direct contact with the bone, potentially increasing bone temperature. This rise in temperature could negatively affect the process of osseointegration (Li et al. 2019).

Therefore, the purpose of this retrospective clinical study was to assess the accuracy of implant positioning, expressed through depth error, mesio‐distal linear deviation, and axial deviation, using a fully digital guided workflow in partially edentulous patients.

## 2 Materials and Methods

This study was designed as a retrospective case series clinical investigation. The study was approved by the Ethical Committee of the University of Naples Federico II (prot. no. 234/21). The analysis was performed on CBCT of patients (both sexes, aged 40–58 years) admitted to the Department of Oral Surgery at the University of Naples Federico II from September 2023 to February 2024, according to the following inclusion criteria:

- healthy patients,
- full mouth bleeding on probing and full mouth plaque index lower than 25%,
- patients requiring one implant or at most two implants in a partial edentulism,
- residual alveolar crest of at least 7 mm length and 5.5 mm width,
- patients with stable teeth close to edentulous region.

Exclusion criteria were the following:

- general contraindications to implant surgery,
- history of radiation to the head and neck region,
- alcohol or drug abuse,
- patients who are pregnant or nursing,
- patients with untreated periodontal conditions and erratic compliance with a supportive periodontal therapy (SPT) program.

All implants were placed using a fully guided implant surgery protocol. A digital workflow was employed to match DICOM and STL files using BlueSkyPlan software (BlueSky Bio, Version 4.7.55, GmbH, Langenhagen, Germany) for preoperative implant placement planning. An ULTIMATE G42 guided surgery kit (GlobalD, France) was utilized for implant placement. Postoperative CBCT scans were conducted for all patients. The deviation between the actual implant position obtained from the postoperative CBCT scan and the planned implant position was assessed. This study was conducted in accordance with the ethical principles outlined in the World Medical Association Declaration of Helsinki for research involving human subjects, and the methods used adhered to the Strengthening the Reporting of Observational Studies in Epidemiology (STROBE) guidelines for observational studies. A detailed description of the surgical treatment was provided, and written informed consent was obtained from each patient (von Elm et al. 2014).

### 2.1 Digital Workflow

**Figure 1:**
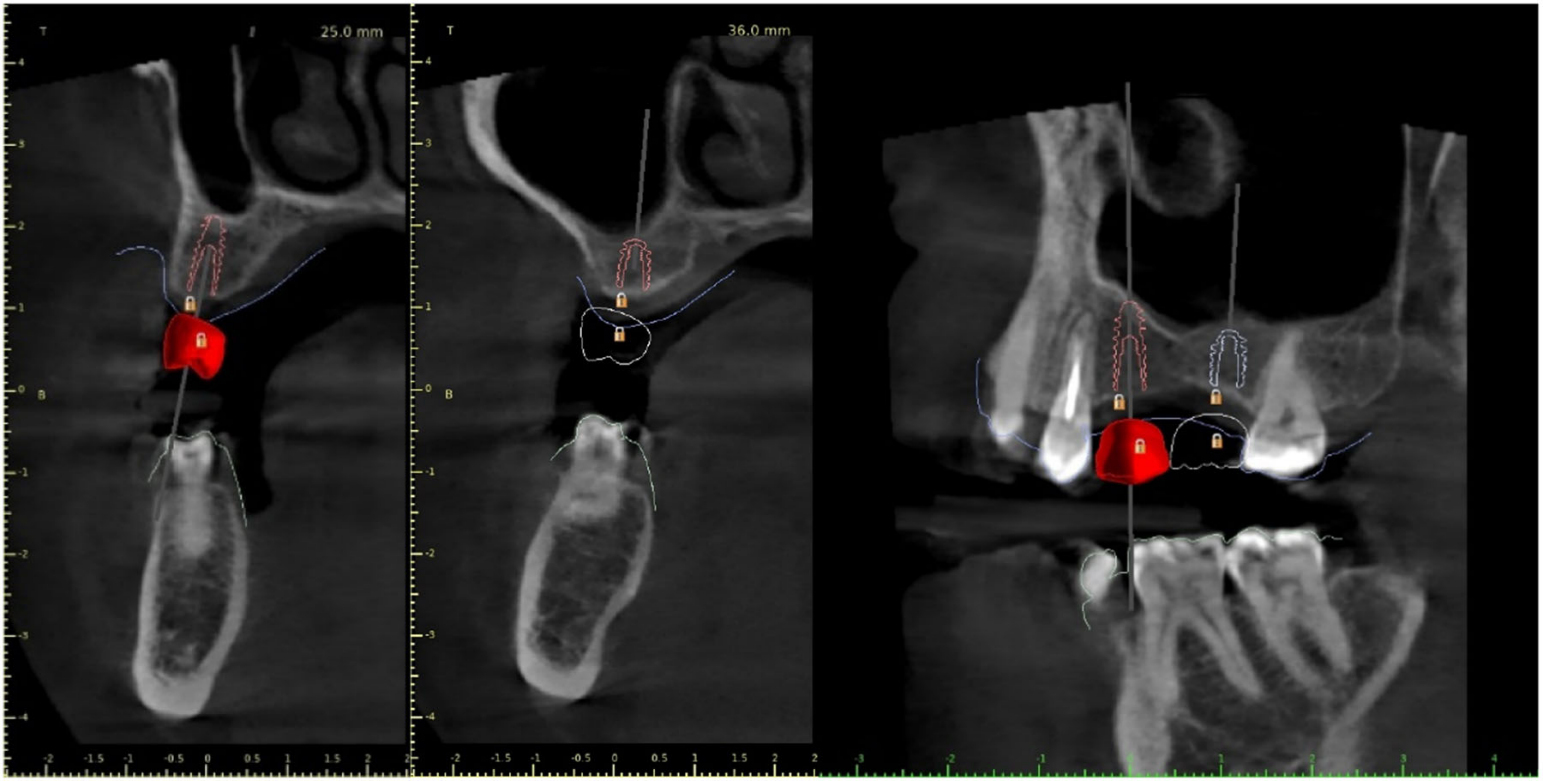
Implant digital planning. The red line indicates the profile of the implant fixture relative to the position of the prosthetic crown.

After the clinical examination, all patients underwent a digital assessment, including a CBCT scan. A high‐speed CBCT scanner (Carestream 8200 3D, Carestream Dental, Germany) was utilized with the following settings: a field of view of 120 mm in height and 10 mm in width, high resolution (voxel size 150), 90 kV, 4 mA, and an exposure time of 20 s. An accurate intraoral scan of both dental arches was obtained using the Helios 600 system (Dentalica Spa, Italy). The prosthetically driven implant positioning was determined through a digital wax‐up (using computer‐assisted design software) of the teeth to be replaced. Finally, the 3D CBCT scan and intraoral scan were superimposed by matching the resulting DICOM and STL data files in BlueSkyPlan software, allowing for the creation of a tooth‐supported surgical guide made of resin, featuring one or two metal sleeves for implant positioning (Figure 1).

### 2.2 Surgical Guide

**Figure 2:**
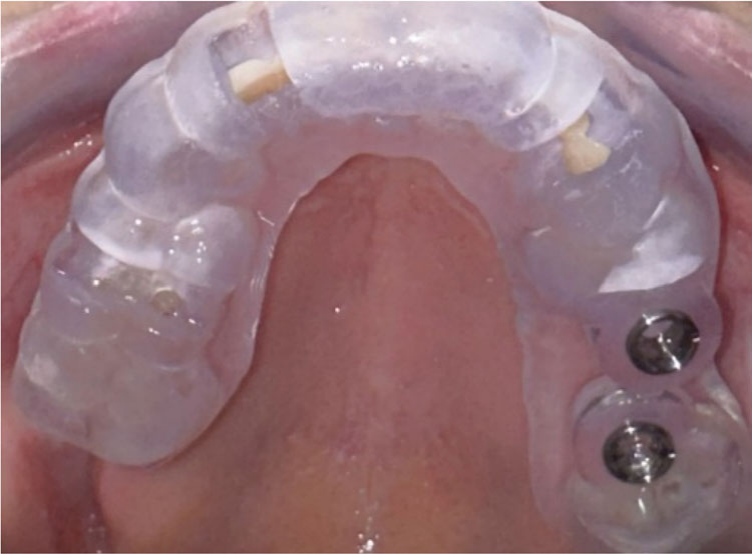
Tooth‐supported surgical guide.

The surgical guide was designed according to the practitioner's implant planning and was precisely manufactured and milled through 3D printing at the premises of the dental surgeon. Titanium guide sleeves with a diameter of 4.2 mm were then inserted into the prepared holes and secured with adhesive, if necessary, in accordance with the planned positioning. Before testing the surgical guide, it was decontaminated by soaking in 4% Gigasept (Gigasept Instru AF; Schülke & Mayer GmbH, Norderstedt, Germany) for 2 h. The tooth‐supported surgical guides were then placed in the patient's mouth, ensuring proper fit. We verified that the guide was stable and retained adequately before proceeding with implant placement (Figure 2).

### 2.3 Surgical Treatment

Before surgery, patients received periodontal treatment including scaling and, if necessary, patients were subsequently root‐planed so that they could achieve a full‐mouth bleeding on probing and full‐mouth plaque index lower than 25%. All surgeries were performed by a single expert surgeon (M.T.). Local anesthesia with 2% mepivacaine 1:100000 adrenaline (Septodont, France) was used. The procedure involved the placement of the implants (Inkone Universal ST, Global D, France) according to treatment plan. In all cases, an adequate amount of keratinized tissue was available (patients who had at least 2 mm of keratinized tissue and a well‐represented vestibular bone cortex of at least 2 mm).

The implant placement was made using the normal sequence of drills 600–800 rpm under external sterile and cold saline solution according to a flapless technique without incisions and suture. A surgical kit for a full‐guided technique (ULTIMATE G42 guided surgery kit, GlobalD, France) was used for implant site preparation until the final insertion of dental fixtures. If immediate loading was scheduled, the provisional screwed retained restorations in PMMA were realized before the surgery and passivated in oral cavity immediately after the implant insertion if the primary stability was at least 40 Ncm. Definitive prosthesis was delivered after 3.5 months in mandible and 4.5 months in upper jaw. Postsurgical instructions included a soft diet and appropriate oral hygiene, avoiding brushing and trauma at the level of surgical sites. Patients were instructed to rinse with a 0.20% chlorhexidine mouth rinse twice a day for about 2 weeks. Antibiotic therapy with amoxicillin + clavulanic acid 1 g twice a day for 6 days was prescribed. The patient was enrolled in an SPT program with regular recall visit every 6 months.

### 2.4 Outcome Measures

The primary outcome was the accuracy of the technique measured as the deviation between the real implant position obtained from the postoperative cone‐beam computed tomography (CBCT) scan and the planned implant position. Two points respectively at the implant shoulder and apex were considered in planned and real position of implants. The superimposition of the CBCTs was performed by the software BluSkyPLan: using the “tangential view tool” in the software in three different positions around implants the depth error, mesio‐distal linear deviation and deviation of angulation degree were evaluated. Descriptive analysis was performed using mean and standard deviation. The secondary outcomes were biological complications, implant failure (implant mobility and/or removal of stable implant due to marginal bone loss or infection) according to the criteria of Misch and collaborators (Misch et al. 2008), prosthetic failure (loss of prosthesis secondary to implant loss, prosthesis replacement for any reason), esthetic outcome, and overall patient satisfaction. The outcomes were evaluated by one independent blinded investigator (A.T.G.).

## 3 Results

Table 1 summarizes the patient and intervention characteristics.

**Table 1:** Patient and intervention characteristics.

Number of patients	21
Mean age (SD)	46.5 (5.81)
Age range	40‐58
Total number of inserted implants	37
Average number of implants in each patient	1.76 (0.62)
Mean diameter of inserted implants (SD)	3.77 (0.25) mm
Mean length of inserted implants (SD)	9,78 (1.30) mm

**Figure 3:**
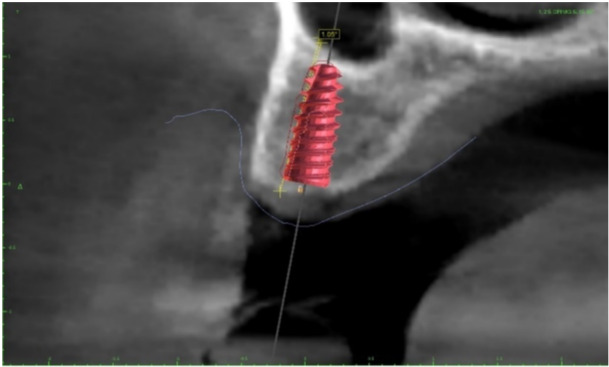
Mesio‐distal deviation measured at the implant shoulder and depth error between the planned and real position of implant. Yellow indicates the profile of implant in real position after surgery, whereas red implant indicates the implant in planned position.

**Figure 4:**
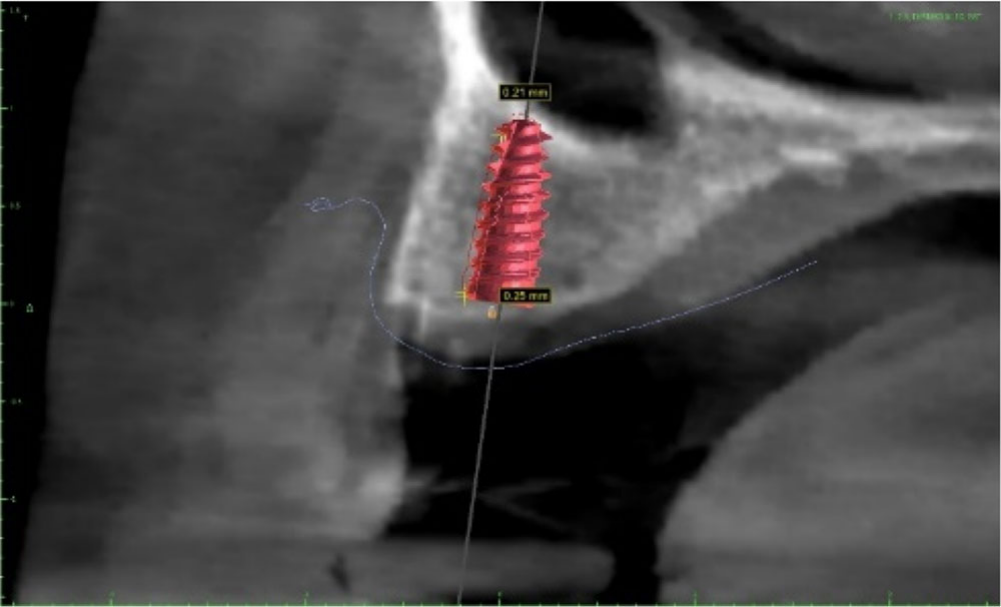
Angular deviation between planned and real position of implant. Yellow indicates the profile of implant in real position after surgery, whereas red implant indicates the implant in planned position.

A total of 21 patients (9 males, 12 females; mean age 46.5 ± 5.81; age range 40–58 years old) were enrolled, and a total of 37 implants were placed. In one female patient and in one male patient, two implants were placed at the level of lower lateral incisors to rehabilitate the four incisors. In only two cases, an immediate loading was made splinting, in provisional restoration, two left lower incisors (in the first case) and two central lower incisors (in the second case). The healing was uneventful for all patients, no complications and no implant or prosthetic failures occurred in a mean of 6 months follow‐up. The average mesio‐distal linear deviation at the implant shoulder was 0.43 ± 0.20 mm and the average depth error was 0.24 ± 0.07 mm (Figure 3). The axis deviation was 1.46° ± 0.31° (Figure 4). All patients confirmed that they were highly satisfied with both the function and esthetic of their implant‐supported prostheses.

## 4 Discussion

The aim of this study was to analyze the accuracy of dental implant placement using a fully digital guided technique. Guided implantology was first introduced by Tomas Fortin in 1995, at Lyon University (France), to address the limitations of traditional free‐hand implant positioning (Fortin et al. 1995). These limitations often resulted in incorrect anatomical and prosthetic insertions, leading to discrepancies between the implant axis and the corresponding prosthetic axis (Bathija et al. 2025). Accurate implant placement using CAIS systems relies on factors like imaging quality, surgical application, and patient characteristics. For imaging error, the layer thickness and voxel sizes contribute to the accuracy (Bathija et al. 2025). Besides, the preoperative and postoperative CBCT need to maintain the same parameters for the deviation measurement. Moreover, different 3D printer technologies affected fully guided implant surgery (Siqueira et al. 2020). The accuracy is currently evaluated after a superimposition of pre‐ and post‐CBCT by analyzing three different parameters: depth deviation, which measures the discrepancy (in millimeters) between the planned and actual implant positions in the vertical plane; angular deviation, which quantifies the angular difference (in degrees) between the planned and actual positions; and global deviation, defined as the metric discrepancy (in millimeters) between the planned and actual positions in the bucco‐lingual and/or mesio‐distal planes relative to the coronal and apical regions of the implant body (Tattan et al. 2020). In this study, the mesio‐distal linear deviation was 0.31 ± 0.10 mm, the angular deviation was 1.21° ± 0.38°, and the depth error was 0.23 ± 0.08 mm. These results are consistent with or slightly lower than those reported in recent systematic reviews (Tahmaseb et al. 2018; Matsumura et al. 2021). For example, a systematic review by Rafael Siqueira et al. found an angular deviation of 2.59° (1.97–3.20) and a depth deviation of 0.55 mm (0.42–0.68 mm) in a fully digital technique involving partially edentulous patients (Matsumura et al. 2021). Another systematic review by Tahmaseb et al. reported a mean error of 0.9 mm [0.79–1.00] for entry point measurement at the center of the implant and 1.2 mm [1.11–1.20 mm] for apical position, with an angular deviation of 3.3° and an average depth error of 0.2 mm [−0.25 to 0.57 mm] for partially edentulous patients (Tahmaseb et al. 2018). Several factors can influence the accuracy of implant positioning (Ngamprasertkit et al. 2022). For instance, greater distances between remaining teeth and the placement site can directly increase angular deviation (Park et al. 2020). The error in implant placement tends to rise with the number of implants being placed in the patient's arch, affecting all three analyzed parameters. Additionally, positioning errors are generally larger in partial‐digital techniques compared to fully digital ones. The characteristics of the surgical guide also play a role: with dental support, the error decreases as the number of supporting teeth increases, particularly becoming minimal when there is a bilateral dental support (Shi et al. 2023). In cases with mucosal support, the number of pins affects angular deviation, with increased pin counts leading to reduced positioning errors (Zhou et al. 2018). The presence of a reinforcing structure in the template also contributes to lower error rates (Park et al. 2020). A prospective cohort study evaluating fully guided implant positioning using a three‐dimensionally printed template with non‐metal sleeves analyzed the accuracy of 187 implants, revealing vertical deviations of 0.65 (0.56–0.75) mm, angular deviations of 3.59° (3.30°–3.89°), and linear deviations of 1.16 (1.04–1.28) mm at the platform and 1.50 (1.36–1.65) mm at the apex. Notably, lower accuracy was observed in implants placed at the third or fourth nearest sites to the most distal tooth compared to those at the first or second nearest sites for linear parameters (Pettersson et al. 2014).

As secondary outcomes, in our study, the healing was uneventful for all patients, no complications and no implant or prosthetic failures occurred. Besides the accuracy of the digital planning in the positioning of the implants, other factors may influence the implant survival rates. Peri‐implant soft tissues play a critical role in maintaining long‐term implant stability (Stefanini et al. 2023). The presence of approximately 2 mm of attached or keratinized mucosa contributes to peri‐implant soft tissue stability, reducing plaque accumulation, inflammation, and the risk of infections, ultimately limiting attachment loss and gingival recession. In our study, the presence of approximately 2 mm of attached or keratinized mucosa around planned implants in all patients, allowed to choose a flapless approach, to minimize invasiveness, to reduce intraoperative trauma, patient discomfort, and postoperative morbidity. On the contrary, the absence of a muco‐periosteal flap can increase the risk of implant mispositioning due to limited visibility of the underlying bone topography. This challenge can be effectively mitigated through pre‐implant planning and the use of a positioning template via the fully digital approach (Del Amo et al. 2020; Romandini et al. 2023). Pre‐implant positioning using computer guidance is essential for accurate placement, considering both hard and soft tissues through a detailed analysis of the patient's anatomy (Battista et al. 2022).

Other factors that influence the survival rate may be the marginal bone loss and the fixture design. The extent of peri‐implant bone resorption is influenced by the type of implant used (Palomo et al. 2008; Stacchi et al. 2023), whereas the choice of implant placement technique appears to have no significant impact. Studies comparing MBL around implants placed using free‐hand versus guided techniques have shown no statistically significant differences (Antonelli et al. 2023). The use of guided implant placement, particularly when combined with flapless surgery, has been associated with better preservation of the alveolar bone around the implant, especially during the 1–3 years follow‐up period (Tallarico et al. 2018; Simonpieri et al. 2017).

Despite the promising results, this study has several limitations that should be acknowledged. First, the small sample size and retrospective design limit the generalizability of the findings. Furthermore, the short follow‐up period and the reduced number of implants evaluated may not be sufficient to assess long‐term outcomes such as marginal bone loss or prosthetic complications. Another important limitation lies in the method of accuracy evaluation, which involved the superimposition of preoperative and postoperative CBCT scans. Different CBCT scans may be obtained using varying protocols, including exposure settings, field of view, and patient positioning. This variability can affect image quality and accuracy, making precise superimposition challenging (Yadav et al. 2018). Moreover, if the two scans are taken at different times, there may be anatomical changes due to healing, growth, or other factors (e.g., bone resorption). These changes can complicate accurate alignment and interpretation of the images. Accurately aligning and superimposing scans requires precise registration algorithms. Differences in imaging parameters or patient movement can lead to errors in registration, affecting the reliability of the planned implant placement. The postoperative CBCT scan may have different levels of artifacts or noise, due to the presence of the implants, as scattering and bone hardening, which can distort the anatomical structures and make it harder to achieve an accurate superimposition (Bruno et al. 2018). This can lead to misinterpretation of critical anatomical landmarks. Moreover, although fully guided implant placement offers high precision, it also has inherent limitations, particularly in cases of severely atrophic jaws. In these scenarios, even minimal deviations in implant positioning may have significant clinical consequences due to limited bone volume and proximity to critical anatomical structures. The rigid nature of surgical guides and potential mucosal compressibility can result in a misfit or displacement of the guide during surgery, which may reduce accuracy. Deviations of even a few millimeters can critically affect treatment success in atrophic ridges, emphasizing the need for additional caution and possibly the integration of alternative techniques such as dynamic navigation or customized support structures in complex cases (Stille et al. 2016; Esposito et al. 2015; Vaira et al. 2024; Esposito et al. 2017).

## 5 Conclusions

Within the limits of the present study, fully guided implant placement can be considered a precise and safe procedure for achieving clinically acceptable positioning accuracy of dental implants in partially edentulous patients. It assists surgeons in accurately positioning implants while respecting both hard and soft tissues, particularly in flapless approaches. However, additional prospective studies with larger sample sizes are necessary to validate these findings.

## Author Contributions

Conceptualization: G.Sa. Data curation: G.Sp. and A.C. Formal analysis: I.F. and A.T.G. Investigation: M.T. Methodology: R.G. Project administration: G.Sa. Resources: M.T. Software: M.T. Supervision: G.Sa. Validation: G.Sp. Visualization: A.C. Writing – original draft preparation: I.F. and A.T.G. Writing – review and editing: R.G.

## Conflicts of Interest

The authors declare no conflicts of interest.

## Data Availability

The data that support the findings of this study are available on request from the corresponding author. G.S. The data are not publicly available due to privacy or ethical restrictions.
